# Willingness to pay for a National Health Insurance Scheme in The Gambia: a contingent valuation study

**DOI:** 10.1093/heapol/czac089

**Published:** 2022-10-27

**Authors:** Hassan Njie, Knut Reidar Wangen, Lumbwe Chola, Unni Gopinathan, Ibrahimu Mdala, Johanne S Sundby, Patrick G C Ilboudo

**Affiliations:** Department of Community Medicine and Global Health, University of Oslo, Postboks 1130 Blindern 0318, Oslo, Norway; Department of Health Management and Health Economics, University of Oslo, Postboks 1089 Blindern, Oslo 0317, Norway; Department of Health Management and Health Economics, University of Oslo, Postboks 1089 Blindern, Oslo 0317, Norway; Norwegian Institute of Public Health, Sandakerveien 24c, Bygg D, Oslo 0473, Norway; Norwegian Institute of Public Health, Sandakerveien 24c, Bygg D, Oslo 0473, Norway; Department of General Practice, University of Oslo, Postboks 1130 Blindern, Oslo 0318, Norway; Department of Community Medicine and Global Health, University of Oslo, Postboks 1130 Blindern 0318, Oslo, Norway; African Population and Health Research Center, APHRC Campus, Manga Close, Off Kirawa Road, P.O. Box 10787-00100, Nairobi 0318, Kenya

**Keywords:** Universal health coverage, health financing, health economics, national health insurance scheme, willingness to pay, contingent valuation, equity

## Abstract

In pursuit of universal health coverage, many low- and middle-income countries are reforming their health financing systems and introducing health insurance schemes. As part of these reforms, lawmakers in The Gambia enacted ‘The National Health Insurance Bill, 2021’. The Act will establish a National Health Insurance Scheme (NHIS) that pays for the cost of healthcare services for its members. This study assessed Gambians’ willingness to pay (WTP) for a NHIS. Using multistage sampling design with no replacement, head/co-head of households were presented with a hypothetical health insurance scheme from July to August 2020. Their WTP and factors influencing WTP were elicited using a contingent valuation method. Descriptive statistics were used to describe sample characteristics. Lopez-Feldman’s modified ordered probit model and linear regression were applied to estimate respondents’ WTP as well as identify factors that influence their WTP. More than 90% of the respondents—677 (94.4%) were willing to join and pay for the scheme. Half of these respondents—398 (58.8%) agreed to pay the first bid of US dollars (US$) 20.78 or Gambian dalasi (GMD) 1000. The average WTP was estimated at US$23.27 (GMD1119.82), whereas average maximum amount to pay was US$26.01 (GMD1251.16). Results of the two models together showed that gender, level of education and household income were statistically significant, with the latter showing negative influence on WTP. The study found that Gambians were largely receptive to the scheme and have stated their willingness to contribute. Our findings can inform policymakers in The Gambia and other sub-Saharan countries when establishing contribution rates and exemption criteria during social health insurance scheme implementation.

## Introduction

The Gambia is the smallest country in mainland Africa with an estimated population of 2.4 million people and an annual growth rate of 3.3%. With an average 8.2 persons per household and 176 people per square kilometre, it is one of the most densely populated countries in Africa (Gambia Bureau of Statistics, 2017a). The Gambia’s economy largely relies on tourism, remittances and rain-dependent agriculture. The 2020 unemployment rate was about 40% and poverty level was estimated at 48.6% (African Development Bank Group, 2021). Following a decline in the economy in 2020 as a result of the SARS- CoV- 2 (COVID-19) global pandemic, the economy is showing signs of slowed recovery (International Monetary Fund, 2021). However, the economic recovery may be impacted negatively due to the ongoing war in Ukraine and increasing food and energy prices globally.

The Ministry of Health (MoH) manages and finances public health care through a theoretically subsidized health system using blended input-based line item and to a lesser degree, programme-based budgeting. User fee charges for Gambians seeking outpatient consultations is pegged at ∼US$0.5 (GMD25) and weekly bed charge of US$2.0 (GMD100). These charges applies to Gambian nationals who are 14 years of age and above, whereas non-Gambians are charged separately. These user fees are managed through the drug revolving fund to supplement pharmaceutical product budget for tertiary care facilities and to a lesser extent, secondary and primary care facilities (Ministry of Health and Social Welfare, 2014; Sine *et al.*, 2019).

Many studies have shown that progress towards universal health coverage (UHC) requires the predominant use of domestic funding to finance health (Reeves *et al.*, 2015; Mathauer *et al.*, 2019). UHC implies that all people have access to needed quality health services (including prevention, promotion, treatment, rehabilitation and palliation) without users being exposed to financial hardship (World Health Organization, 2010a). However, this is not the case in The Gambia, where general government health expenditures domestic, as percentage of gross domestic product (GDP), was 1% in 2019 (World Health Organisation, 2022). This represents less than the recommended threshold of government spending of at least 5% of GDP on health (McIntyre *et al.*, 2017). The most recent National Health Account (NHA) in The Gambia has shown that the current health expenditure per capita was US$25.84 (Ministry of Health, 2020). This falls short of the Commission on Macroeconomics and Health estimates that by 2015, low- and middle-income countries (LMICs) should spend at least US$71 on health, whereas High-Level Taskforce estimated per-capita spending on health of US$86—all expressed in 2012 US$ terms (McIntyre *et al.*, 2017). Recent estimates also show that LMICs should spend at least US$76 per person per year to build sustainable and resilient health system and make progress towards UHC (Stenberg *et al.*, 2017). Furthermore, the NHA findings show that, as percentage of current health expenditure, general government health expenditure was 27.20%, external funding was 45.49% and out-of-pocket (OOP) spending was 26.96% (Ministry of Health, 2020). These estimates show that Gambia’s health financing is heavily dependent on donor funding.

Many LMICs are exploring various health financing mechanisms, including social health insurance schemes to offer financial protection to their populations (Ogundeji *et al.*, 2019). As part of UHC reforms, the National Assembly of The Gambia in 2021 enacted into law, ‘The National Health Insurance Bill, 2021’ (Ministry of Health, 2021). The Act will establish a mandatory National Health Insurance Scheme (NHIS) that will pay for the cost of healthcare services to members of the scheme. This development in The Gambia aligns with global efforts to achieve UHC (World Health Organization, 2010a). The success of the NHIS is dependent on the support and involvement of the public in this much-needed public policy reform. Considering public perceptions and preferences when designing its health financing system plays a crucial role in creating a sustainable health insurance scheme.

Previous studies have shown that communities have clear preferences for their healthcare needs when asked to contribute (Nguyen *et al.*, 2017). Little is known, however, about support for health financing reforms and in particular public preferences for NHIS in The Gambia. Many studies have reported that social health insurance schemes increase access and utilization of health services, thereby propelling countries towards UHC (Alhassan *et al.*, 2016; Dalinjong *et al.*, 2017; Van der Wielen *et al.*, 2018; Bodhisane and Pongpanich, 2019; Erlangga *et al.*, 2019; van Hees *et al.*, 2019). Others, however, reported that they do not protect against financial risks, but rather increase inequities in health particularly among underserved and vulnerable populations (Kotoh and Van der Geest, 2016; Prinja *et al.*, 2017; Okoroh *et al.*, 2018). The latter is particularly true for countries implementing community-based health insurance schemes, where risk pooling potential is reduced due to its voluntary pre-payment design.

Against this background, our study had two primary objectives: first, to estimate the willingness to pay (WTP) for NHIS in The Gambia. Second, to identify factors associated with different levels of WTP as well as explored reasons for Gambians’ unwillingness to join and pay for NHIS. Our study can translate evidence-based research into effective planning and policymaking. This study is important for policymakers in The Gambia and other sub-Saharan African countries to set progressive contribution rates and exemption criteria that maximize the number of citizens to benefit from the NHIS.

## Materials and methods

### Study setting

This study was conducted in The Gambia between July and August 2020. We utilized a nationally representative cross-sectional survey using a contingent valuation (CV) method to elicit Gambians’ WTP in a hypothetical NHIS. This study received ethical clearance from The Gambia Government/Medical Research Council Joint Ethics Committee (R018026v4.1) and Norwegian Centre for Research Data (562 557). The Norwegian Research Committee for Medical and Health Research Ethics exempted the study from ethical reviews (2018/1891).

### Sampling approach

The Gambia Bureau of Statistics (GBoS) demarcates the country into 4098 enumeration areas (EA) or clusters. Each EA (cluster) comprises 500 people, whereas in smaller communities, two or three villages are combined to constitute one EA (cluster). The 2013 population and housing census estimated 280 702 households in The Gambia (Gambia Bureau of Statistics, 2018b).

**Figure 1.**
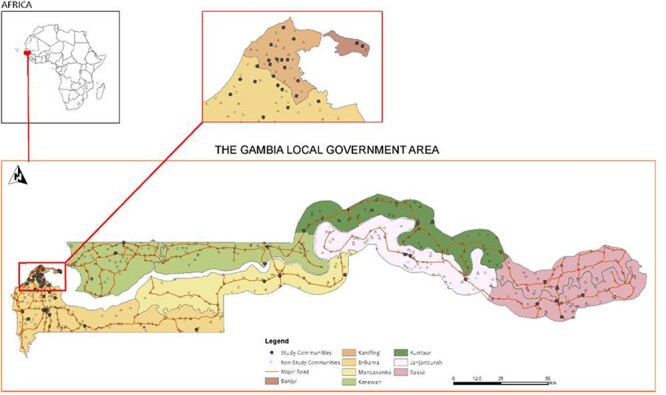
Distribution of study communities across The Gambia

We used a two-stage sampling design without replacement as described by Elfil and Negida (Elfil and Negida, 2017). In the first stage, clustered EAs were systematically sampled using probability proportionate to size technique. Following the first stage sampling, teams of enumerators were deployed to the sampled EAs to identify and assign numbers to eligible households for selection. In the second stage, households were systematically sampled proportional to the number of households in each EA using the multiple indicator cluster survey (MICS6) systematic random selection template adapted for this study (Gambia Bureau of Statistics, 2018b). Finally, eligible household heads/co-heads were selected for an interview. The numbered household list generated during the first stage sampling was the sampling frame. Figure 1 shows distribution of study communities.

The MICS6 systematic random selection template adapted for this study was validated and used in 2018 Gambia multiple indicator cluster survey, 2019 Gambia demographic and health survey and 2020 Gambia integrated household survey. From these population data, we selected a sample size of 780 respondents. We used a conservative assumption that 50% is the proportion of respondents who were willing to pay for NHIS. With 80% desired statistical power, a significance level alpha of 0.05 and a margin of error of 0.04, the minimum sample size estimated was 600. However, since this is a clustered survey with EAs acting as clusters, the sample size was inflated using a design effect of 1.3 to give a minimal sample size of 780.

Gambian nationals 18 years of age and above, who were heads/co-heads of households and have consented to participate in the study were included. Non-Gambian residents were excluded.

### Study instrument

Interviewer-administered questionnaires designed according to the CV guideline were used to collect relevant information from the respondents (Bateman *et al.*, 2002). The questionnaire was later validated internally by a pool of researchers familiar with CV studies. A pre-test of the questionnaire among 30 individuals was done in two phases prior to data collection. The questionnaire was refined for clarity and ease of comprehension following the pre-test.

Research assistants were recruited mainly from the University of The Gambia and the MoH. The recruitment criteria included background in any of the following disciplines: nursing, public health, biostatistics or experience in health surveys. Those recruited underwent 2 days of training on the conduct of cross-sectional survey and administration of the questionnaire. At the end of the training, enumerators pre-tested the questionnaire.

### Variable specification and priori expectation

The outcome variable for our study was Gambians’ WTP for NHIS. In our study, this is defined as WTP, a dummy variable with 1 denoting an individual’s WTP and 0, otherwise. Explanatory variables selected for our study were adapted from a systematic review of WTP for health insurance in LMICs (Nosratnejad *et al.*, 2016). These variables were divided into two parts: demographic and socio-economic characteristics and health service characteristics including private insurance coverage. Variable specification and priori expectation are in Table 4.

Existing studies have shown that males, young adults, larger households, low-income households, higher education, previous hospitalization and perceived poor health status influenced respondents’ WTP (Nosratnejad *et al.*, 2016; Al-Hanawi *et al.*, 2018). Therefore, we hypothesized that in The Gambia, males, higher education, previous hospitalization and perceived poor health had a higher WTP. Young adults, larger households and low-income households had lower WTP. Table 4 shows variable specification and priori expectation.

### Eliciting WTP

We used the CV method applying the double-bounded dichotomous choice (DBDC) questions with follow-up approach as described by Hanemann (Hanemann, 1989) and Lopez-Feldman (Lopez- Feldman, 2012). CV is widely used to assess WTP changes in non-marketed goods such as health insurance (Gidey *et al.*, 2019; Ogundeji *et al.*, 2019). DBDC formats have been shown to have the greater efficiency as they enable respondents to disclose more information on their WTP (Hanemann *et al.*, 1991). A description of the DBDC model equation is provided in Supplementary materials Part 1.

To ascertain respondents’ WTP for NHIS, an overview of the current health financing situation in The Gambia was presented to them. This was done to ensure that they understood the current financing situation and to inform an objective response to the hypothetical NHIS scenario.

**Figure 2.**
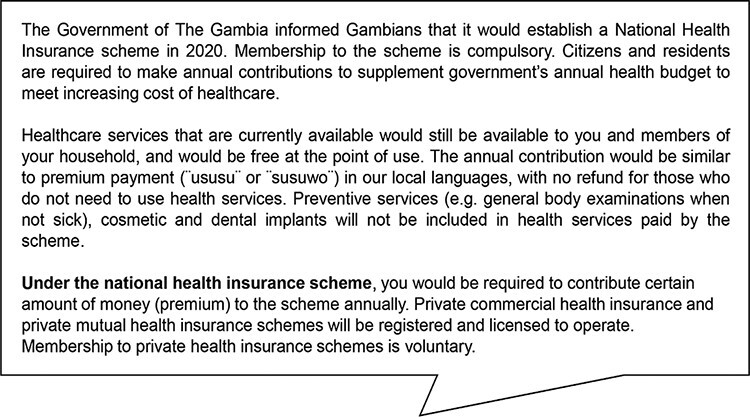
Hypothetical contingent market (valuation scenario)

Following the description of the hypothetical contingent market, as depicted in Figure 2, respondents were asked whether they were willing to join and pay for the scheme. Those that agreed were offered the first bid, which is the specific price of the non-marketed commodity in question, of US$20.78 (GMD1000). If they answered yes to the first bid, the upper bid of US$31.17 (GMD1500) was offered and if they answered no to the first bid, a lower bid of US$10.39 (GMD500) was offered. We increased the first bid by half for the upper bid and reduced the first bid by half for the lower bid. All monetary estimates were expressed in current US$ although the amounts presented to the respondents were in GMD. All respondents that agreed to join and pay for NHIS were asked to state the maximum amount they were willing to pay with no restriction if the government was to introduce NHIS on the day of the data collection. Respondents that refused to join the scheme were asked to state their reasons. The starting bid amount was determined by using the average WTP as percentage of GDP per capita (2.97) of nine WTP studies conducted in West Africa based on systematic review of WTP for health insurance in LMICs (Nosratnejad *et al.*, 2016).

### Statistical analysis

We applied Lopez-Feldman’s econometric specification for the double-bounded model, conferred Supplementary materials Part 1 and used the maximum-likelihood method for the estimation. He described this as a modified ordered probit model, otherwise known as doubleb command in Stata (Lopez- Feldman, 2012). We estimated the WTP of participants by using the average values of the explanatory variables included in the model. Separately, we used linear regression to estimate the average maximum amount to pay as well as explore the relationship between respondents’ response and explanatory variables. The equation for the regression model is in Supplementary materials Part 2. Descriptive analysis presents respondents’ demographic, socio-economic and health service characteristics as shown in Table 1

**Table 1.** Demographic, socio-economic and health service characteristics

	All (*n*, %)	Urban (*n*, %)	Rural (*n*, %)
Local government area (*N*, %)	717 (100.0)	468 (65.3)	249 (34.7)
Gender			
Female	391 (54.5)	284 (39.6)	107 (14.9)
Male	326 (45.5)	184 (25.7)	142 (19.8)
Age (in years)			
≤30	179 (25.0)	127 (17.7)	52 (7.3)
31–40	189 (26.4)	134 (18.7)	55 (7.7)
41–55	201 (28.0)	130 (18.1)	71 (9.9)
>55	148 (20.6)	77 (10.7)	71 (9.9)
Marital status			
Never married	29 (4.0)	20 (2.8)	9 (1.3)
Married	647 (90.2)	415 (57.9)	232 (32.4)
Living together, divorced, separated, widow	41 (5.7)	33 (4.6)	8 (1.1)
Education			
Low (no formal and primary education)	474 (66.1)	268 (37.4)	206 (28.7)
Middle (junior and senior secondary, vocational, professional)	226 (31.5)	185 (25.8)	41 (5.7)
Higher (university degree and above)	17 (2.4)	15 (2.1)	2 (0.3)
Employment			
Not in employment, retired	312 (43.5)	203 (28.3)	109 (15.2)
Public or private sector employee	274 (38.2)	194 (27.1)	80 (11.2)
Informal sector	131 (18.3)	71 (9.9)	60 (8.4)
Household size			
1–7 persons	229 (31.9)	187 (26.1)	42 (5.9)
8–15 persons	280 (39.1)	193 (26.9)	87 (12.1)
≥16 persons	208 (29.0)	88 (12.3)	120 (16.7)
*Household monthly income (in GMD)			
<GMD500.00—GMD9999.00	698 (97.4)	454 (63.3)	244 (34.0)
GMD10 000.00—GMD19 999.00	17 (2.4)	13 (1.8)	4 (0.6)
≥GMD20 000.00	2 (0.3)	1 (0.1)	1 (0.1)
*Access to health facility			
No	64 (9.0)	33 (4.6)	34 (4.7)
Yes	650 (91.0)	435 (60.7)	215 (30.0)
Outpatient visit (last 12 months)			
0 visit	153 (21.3)	106 (14.8)	47 (6.6)
1–3 visits	255 (35.6)	188 (26.2)	67 (9.3)
≥4 visits	309 (43.1)	174 (24.3)	135 (18.8)
Expensed on outpatient visit including medicines in last 12 months (in GMD)			
GMD0.00	459 (64.0)	306 (42.7)	153 (21.3)
GMD1.00—GMD504.00	173 (24.1)	118 (16.5)	55 (7.7)
≥GMD505.00	85 (11.9)	44 (6.1)	41 (5.7)
Hospitalization (last 12 months)			
0 hospitalization	691 (96.4)	459 (64.0)	232 (32.4)
≥1 hospitalization	26 (3.6)	9 (1.3)	17 (2.4)
Expensed on hospitalization including medicines in last 12 months (in GMD)			
GMD0.00	691 (96.4)	459 (64.0)	232 (32.4)
GMD1.00—GMD504.00	14 (2.0)	5 (0.7)	9 (1.3)
≥GMD505.00	12 (1.7)	4 (0.6)	8 (1.1)
First point of care is traditional/spiritual/herbal medicine			
No	484 (67.5)	350 (48.8)	134 (18.7)
Yes	233 (32.5)	118 (16.5)	115 (16.0)
Expensed on traditional/spiritual/herbal medicines in last 12 months (in GMD)			
GMD0.00	484 (67.5)	350 (48.8)	134 (18.7)
GMD1.00—GMD504.00	164 (22.9)	83 (11.6)	81 (11.3)
≥GMD505.00	69 (9.6)	35 (4.9)	34 (4.7)
Presence of chronic disease			
No	501 (69.9)	341 (47.6)	160 (22.3)
Yes	216 (30.1)	127 (17.7)	89 (12.4)
Perceived state of health in last 24 h			
Poor	24 (3.3)	13 (1.8)	11 (1.5)
Fair	181 (25.2)	115 (16.0)	66 (9.2)
Good, very good, excellent	512 (71.4)	340 (47.4)	172 (24.0)
Perceived level of satisfaction with health services			
Unsatisfied	194 (27.1)	141 (19.7)	53 (7.4)
Do not know	39 (5.4)	28 (3.9)	11 (1.5)
Satisfied	484 (67.5)	299 (41.7)	185 (25.8)
Private health insurance coverage			
No	682 (95.1)	441 (61.5)	241 (33.6)
Yes	35 (4.9)	27 (3.8)	8 (1.1)

* Household monthly income in (GMD)= adjusted relative to household size using the equivalence scale developed by Swiss Conference of Social Assistance.

* Access to health facility measurement= not >5 km radius of settlement.

## Results

### Demographic and socio-economic characteristics

Table 1 shows the demographic, socio-economic and health service characteristics of the respondents. Overall, 391 (54.5%) of respondents were females and 368 (51.3%) were between 18 and 40 years of age; 468 (65.3%) of respondents resided in urban areas and 647 (90.2%) were married; 474 (66.1%) had no formal education or stopped at primary school. A total of 274 (38.2%) were in formal employment (public or private), whereas 43.5% were either not in employment or retired. Among all respondents, 208 (29.0%) had household size of 16 or more members, while the corresponding proportion was 16.7% for respondents in rural areas. A majority of the respondents reported that their monthly household income, adjusted for household size, was below the national and international poverty line (<US$1.90 or <GMD26.20 per day) corresponding approximately to <GMD10 000.00 in Table 1. There was no big divide in household income between urban and rural Gambia relative to income class groups above the national or international poverty line.

### Health service characteristics

Overall, 650 of the respondents (91.0%) reported having access to a healthcare facility; 78.7% reported having at least one outpatient visit in the preceding year, and 11.9% reported spending at least US$10.39 (GMD505) on outpatient visits including medicines. Twenty-six respondents (3.6%) experienced hospitalization at least once in the year preceding the survey. Less than 2% of hospitalized respondents reported spending >US$10.39 (GMD505) on bed fees including medicines. Overall, 233 (32.5%) reported that their first point of care was a traditional, herbal or spiritual healer, out of which 69 (29.6%) spent >US$10.39 (GMD505) on these services. There was no major difference between respondents that sought traditional, herbal or spiritual care in urban or rural areas. About a third (30.1%) reported having one or more chronic conditions. Over 70% of respondents perceived that they were in good state of health and two-thirds reported satisfaction with health service delivery. Less than 5% reported having private health insurance. The reporting period was last 12 months preceding the survey.

### Respondents WTP for NHIS

**Figure 3.**
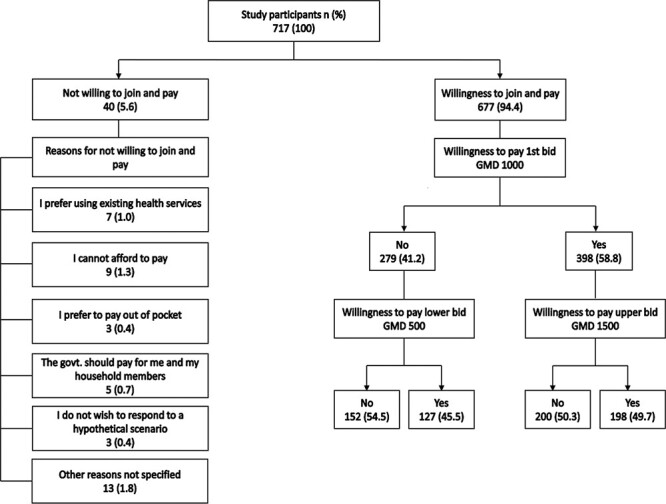
Characteristics of respondents’ WTP or not

Figure 3 shows that 94% of respondents were willing to join and pay for NHIS. Out of this number, about 59% accepted the first bid and ∼50% were willing to pay the upper bid. Out of the almost 6% that refused to join and pay for NHIS, 1% preferred using the existing health services or paying for these services OOP. About 1% reported that they could not afford to pay or preferred the government pay for them and members of their household. The remaining 2% did not wish to respond to a hypothetical scenario or had not specified reasons for not willing to join and pay for NHIS.

Table 2 shows that respondents’ average WTP was US$23.27 (GMD1119.82) [confidence interval (CI): 692.61 to 1547.02]. Males were willing to pay US$5.10 (GMD245.38) more compared with females (CI: 114.09 to 376.68; *P*-value < 0.01).

**Table 2.** Results of estimation of DBDC model

	β (95% CI)	*P*-value
Gender		
Female	Ref.	
Male	245.38 (114.09, 376.68)	<0.01
Age (in years)		
≤30	Ref.	
31–40	−9.72 (−178.71, 159.28)	0.91
41–55	−46.78 (−222.19, 128.63)	0.60
>55	−62.01 (−259.79, 135.76)	0.54
Education		
Low (no formal and primary education)	Ref.	
Middle (junior and senior secondary, vocational, professional)	254.79 (118.88, 390.69)	<0.01
Higher (university degree and above)	208.91 (−179.53, 597.35)	0.29
Household size		
1–7 persons	Ref.	
8–15 persons	85.03 (−58.97, 229.03)	0.25
≥16 persons	152.23 (−7.33, 311.80)	0.06
Household monthly income (in GMD)		
Lower- and upper-middle-income poverty line	Ref.	
Below poverty line	−280.01 (−677.20, 117.21)	0.17
Hospitalization (last 12 months)		
0 hospitalization	Ref.	
≥1 hospitalization	−53.70 (−367.04, 259.65)	0.74
Perceived state of health (in last 24 h)		
Good, very good, excellent	Ref.	
Poor	148.12 (−177.42, 473.65)	0.37
Fair	35.76 (−104.67, 176.18)	0.62
Mean WTP value (in GMD)	1,119.82 (692.61, 1547.02)	<0.01

*P*-value of 0.00 ≤ 0.01. β coefficient is derived from equation in supplementary materials part 1. Ref. = Reference category.

US$ to GMD exchange rate (July–August 2020): 1 US$ = 48.13.

Gambia national poverty line or international poverty line.

Below poverty line = 26.2 in GMD (2015) or US$1.90 (2011 PPP) per day per capita.

Lower-middle-income class poverty line = 44.2 in GMD (2015) or US$3.20 (2011 PPP) per day per capita.

Upper-middle-income class poverty line = 75.9 in GMD (2015) or US$5.50 (2011 PPP) per day per capita.

Respondents with middle education had a WTP of US$5.29 (GMD254.79) higher than the reference group with low education (CI: 118.88 to 390.69; *P*-value < 0.01). The corresponding estimate for the high education group was not significant.

Many estimates had wide CIs, and the observed relationships did not meet the pre-specified threshold for statistical significance; the strength of these relationships therefore carries high uncertainty. Smaller households were observed to have lower WTP US$1.77 (GMD85.03) relative to larger households when compared with the lowest household size (CI: −58.97 to 229.03; *P*-value, 0.25). Respondents below the poverty line had a lower WTP US$ −5.82 (GMD −280.01) compared with the higher-income group (CI: −677.20 to 117.21; *P*-value, 0.17). Respondents that had experienced hospitalization at least once were associated with lower WTP US$ −1.12 (GMD −53.70) compared with those with no history of hospitalization in the preceding year (CI: −367.04 to 259.65; *P*-value, 0.74). Compared with respondents with perceived good health, respondents who perceived their health status as poor were more likely to pay more US$3.08 (GMD148.12) relative to those whose health status was perceived fair (CI: −177.42 to 473.65; *P*-value, 0.37).

Table 3 shows the results of the linear regression model estimating the adjusted average maximum amount to pay for NHIS. Unlike the DBDC model in Table 2, we only present explanatory variables that were statistically significant. Males were more likely to have a higher maximum amount to pay than females by US$4.49 (GMD216.10, CI: 92.64 to 339.57; *P*-value < 0.01). Respondents with middle education were associated with a lower maximum amount to pay US$3.01 (GMD144.90) relative to the higher education category when compared with lower education group (CI: 17.72 to 272.08; *P*-value < 0.01). Respondents below the poverty line were associated with lower maximum amount to pay by US$ −16.90 (GMD −813.30) compared with the higher-income group (CI: −1174.13 to −452.38, *P*-value < 0.01).

**Table 3.** Results of linear regression (generalized linear model)

	β (95% CI)	*P*-value
Gender		
Female	Ref.	
Male	216.10 (92.64, 339.57)	<0.01
Age (in years)		
≤30	Ref.	
31–40	38.83 (−120.46, 198.11)	0.63
41–55	−122.10 (−286.83, 42.62)	0.60
>55	−115.66 (−301.46, 70.13)	0.22
Education		
Low (no formal and primary education)	Ref.	
Middle (junior and senior secondary, vocational, professional)	144.90 (17.72, 272.08)	0.03
Higher (university degree and above)	244.83 (−119.30, 609.01)	0.19
Household size		
1–7 persons	Ref.	
8–15 persons	86. 04 (−50.20, 222.28)	0.22
≥16 persons	83.30 (−66.90, 233.41)	0.28
Household monthly income (in GMD)		
Lower- and upper-middle-income poverty line	Ref.	
Below poverty line	−813.30 (−1174.13, −452.38)	<0.01
Hospitalization (last 12 months)		
0 hospitalization	Ref.	
≥1 hospitalization	37.90 (−259.01, 334.70)	0.80
Perceived state of health (in last 24 h)		
Good, very good, excellent	Ref.	
Poor	15.42 (−291.15, 322.01)	0.92
Fair	26.75 (−105.20, 158.70)	0.69
β_0_-	1,251.16 (860.20, 1642.12)	<0.01

*P*-value of 0.00 ≤ 0.01. β intercept is derived from GLM in Supplementary materials part 2. Ref. = Reference category.

US$ to GMD exchange rate (July–August 2020): 1 US$ = 48.13.

Gambia national poverty line or international poverty line.

Below poverty line = 26.2 in GMD (2015) or US$1.90 (2011 PPP) per day per capita.

Lower-middle-income class poverty line = 44.2 in GMD (2015) or US$3.20 (2011 PPP) per day per capita.

Upper-middle-income class poverty line = 75.9 in GMD (2015) or US$5.50 (2011 PPP) per day per capita.

**Table 4.** Explanatory variable specification and priori expectation

Variables		Explanation	Measurement	Priori expectation
Socio-economic characteristics	Gender	Whether respondent is female or male	0 = female 1 = male	Males are highly likely to pay more than females
	Age	Age of respondent in years	0 ≤ 30 1 = 31–40 2 = 41–55 >55	Young age groups are less likely to pay more compared with other age groups
	Level of education	Level of education attained	0 = No formal and primary 1 = Junior and senior secondary, vocational 2 = University	People with higher education are highly likely to pay more compared with other groups
	Household size	Number of people in a household	0 = 1–7 1 = 8–15 2 ≥ 16	Larger households are less likely to pay more compared with other groups
	Household income	Household income (GMD) adjusted relative to household size	0 ≤ 500–9,999 1 ≥ 10,000	Households with lower income are less likely to pay more compared with higher-income groups
Health service characteristics	Hospitalization	Past experience of hospitalization in last 12 months	0 = No 1 = Yes	People who experience hospitalization are highly likely to pay more compared with other groups
	Perceived state of health	Overall state of health in last 24 h	0 = Poor 1 = Fair 2 = Good	People with perceived poor health are likely to pay more compared with other groups

## Discussion

Public health care in The Gambia is theoretically highly subsidized by the government (Ministry of Health and Social Welfare, 2012). However, evidence suggests that the public health sector remains seriously underfunded with the government unable to allocate at least 5% of GDP to health, a threshold considered by the World Health Organization and health financing experts as minimum domestic funding on health to make progress towards UHC (McIntyre *et al.*, 2017; Ministry of Health and Social Welfare, 2017; Sine *et al.*, 2019). In its attempt to move towards UHC, the government introduced a mandatory NHIS that would pay the cost of health care for Gambians and non-Gambian residents. In view of this major public policy shift, our study estimated Gambians’ willingness to join and pay for a NHIS. The high willingness to join and pay for NHIS could be perceived as high public support to reform health financing in The Gambia. Our finding is consistent with similar findings of WTP studies conducted in LMICs, particularly in countries comparable to The Gambia (Djahini-Afawoubo and Atake, 2018; Jofre-Bonet and Kamara, 2018; Ogundeji *et al.*, 2019). Other studies have also shown that risk-averse individuals tend to opt for insurance coverage to reduce the impact of potential catastrophic risks (Schmitz, 2011; Nguyen *et al.*, 2017).

In our study, individuals were willing to pay on average US$23.27 (GMD1119.82) to join the scheme, which was closer to the first bid of US$20.80 (GMD1000). This finding aligns with previously conducted contingent market valuation of health insurance contributions that shows an inverse relationship between price and acceptance rate, where individuals that accepted first price are less willing to pay more when the price is increased (Nosratnejad *et al.*, 2014; Nguyen and Hoang, 2017). However, there is evidence suggesting that the DBDC with a follow-up model is sensitive to starting point bias (Flachaire and Hollard, 2006; Jofre-Bonet and Kamara, 2018), and we discuss the implications of this under discussion and limitations.

The weak state of public health care in The Gambia could be a key factor for explaining Gambians’ WTP more than the first bid in return for better healthcare services. Evidence suggests that health facilities experience frequent stock out of essential medicines and supplies. Due to limited specialist services including access to advanced health technologies particularly in the public sector, many patients are forced to seek expensive overseas medical treatments in Senegal, India and Turkey (Radio France International, 2018; Sine *et al.*, 2019; Manneh, 2021). The strong statement of intent to pay more for NHIS in return for better health care is a policy window for the government to introduce a scheme that enhances access to quality, affordable and equitable health services. Notwithstanding, it is important to note that higher WTP for NHIS as shown in our study does not equal ability to contribute to the scheme due to possibility loss of incentive compatibility. Given Gambia’s high poverty and unemployment rate, in addition to high informality of the economy, the government should increase domestic revenue-raising capacity including designing robust strategies to increase revenue from indirect taxes to sustainably fund and manage the scheme.

The DBDC model and generalized linear model (GLM) together showed that gender, level of education and household income were associated with Gambians’ WTP and maximum amount to pay for NHIS. The DBDC model in Table 2 shows that respondents’ WTP was significantly influenced by their gender and level of education, whereas the regression model showed that household income was associated with maximum amount to pay, which was statistically significant.

Our finding showed that males were more likely to pay more for NHIS than females as hypothesized. This finding is consistent with observations in similar studies reporting that females have a lower WTP compared with men (Dong *et al.*, 2004; Onwujekwe *et al.*, 2009; Djahini-Afawoubo and Atake, 2018). Gambia is known for its strong patriarchal leaning with men perceived to be head of households and purported ‘bread-winners’ of the family (Bellagamba, 2013). This deeply held belief coupled with limited implementation of gender empowerment policies reduces opportunities for women’s participation in the formal workforce, disproportionately affecting them economically, socially, politically and health wise (Fourshey, 2019; United Nations Capital Development Fund, 2019). For example, the 2015 integrated and household survey reported that females constituted a higher proportion of the working age group but were less economically active than males (Gambia Bureau of Statistics, 2017a). However, the WTP estimated among females could still indicate a high financial commitment to the scheme considering their limited economic opportunities. From the policy perspective, the government should consider providing more opportunities for women’s participation in economic activities. This could increase their WTP because women’s utilization of healthcare services in The Gambia is higher than men (Ministry of Health, 2019). A mapping and analysis of the laws of The Gambia from a gender perspective in 2020 found laws or provisions that prevent women and girls from realizing their full social, cultural, economic, political and civil rights (UN Women and Commonwealth Secretariat, 2020). These laws and implicit policy biases towards women and girls also increases their vulnerabilities (ECOWAS Commission, 2018). In addition to creating economic opportunities for women, the government should also consider widening the social safety net in the scheme. Although the Gambia NHIS Act proposed exemption from premium contribution for pregnant and post-partum women, the exemption criteria should be expanded to include women in lower socio-economic groups and in other disadvantaged positions. This would enhance positive health outcomes for women and girls and reduce gender health inequities in The Gambia.

Compared with respondents with low education, both respondents with middle education and respondents with high education had a higher WTP, although the latter estimate was not statistically significant. Although this is in contrast to our hypothesis, it is important to note that respondents with university education constitute a smaller proportion of the sample in our study. Although few studies are in agreement with our findings (Gidey *et al.*, 2019; Ogundeji *et al.*, 2019), others have shown that individuals with higher education were likely to pay more for health insurance in LMICs (Nosratnejad *et al.*, 2014; Al-Hanawi *et al.*, 2018; Djahini-Afawoubo and Atake, 2018). A plausible explanation for our finding could be that Gambians with higher education were more likely to pay OOP or use private health services including private health insurance to access better services than what they perceive is possible through the public health sector. This can probably be explained by their ability to get a good job and earn higher income than their corresponding groups. An education sector public expenditure review in The Gambia have shown that Gambians with higher education prefer working in private services sectors due to strong employment opportunities and attractive salaries and incentives (The World Bank, 2017).

The financial viability of NHIS will depend on requiring more people to contribute to the scheme than exempted. However, a crucial challenge to the sustainability of The Gambia’s NHIS is the high poverty and unemployment rate especially among the productive age groups (Gambia Bureau of Statistics, 2018a). Similarly, Gambia has one of the highest age dependency ratios in the world, coupled with a household size averaging seven members (Gambia Bureau of Statistics, 2017b). This makes it even more difficult to raise revenue from households with low incomes. Imposing premiums on the poor, formal and informal workers within the low-income bracket is likely to increase financial burden at individual and household levels, thereby increasing vulnerabilities and widening inequities in health. The Gambia is ranked among countries with the lowest minimum wage in the world (Public Administration International, 2020). Thus, heavy reliance on formal and informal sector payroll taxes to finance the scheme without equity considerations such as mean tested approach could challenge the sustainability of NHIS. The government should therefore consider using progressive contribution rates for formal and informal sector workers through means testing, wherein higher-income groups contribute a higher percentage of their incomes to the scheme relative to lower-income groups (McIntyre and Kutzin, 2016). Gambia’s informal sector is huge and accounts for ∼58% of GDP and constitutes almost 77% of total employment (Oladipo, 2021). Despite the Act stipulating mandatory contribution for employees including informal workers, weak enforcement of tax laws and current revenue collection mechanism in the informal sector make it difficult to collect sufficient revenue from this diverse sector. The sustainability of the scheme will depend on domestic revenue-raising capacity and increased domestic funding for health. The informal sector offers strong revenue-raising opportunities, and to efficiently tap into this sector, the government should create an enabling environment for the informal sector to organize formally (Oladipo, 2021). In addition, the government should design a benefit package that is explicit to enhance enrollees’ utility.

The two models combined have shown that age, household size, history of hospitalization and perceived state of health were not statistically significantly and did not influence respondents’ WTP and maximum amount to pay. Many studies in LMICs agree with our finding (Shafie and Hassali, 2013; Nosratnejad *et al.*, 2014; 2016; Al-Hanawi *et al.*, 2018; Gidey *et al.*, 2019). However, other studies have shown that household income did not influence WTP (Nguyen and Hoang, 2017). This finding poses a policy dilemma for the government in its effort to introduce premium rates and exemption criteria in the face of the country’s weak macro-economic outlook. Gambia’s GDP per-capita growth rate was not consistent over the years relative to its aspiration peers (United Nations, 2020b) and has a lower GDP per capita than sub-Saharan Africa average (Gil-Alana *et al.*, 2021). The COVID-19 pandemic continues to exert pressure on the economy due to huge revenue loss from tourism and vital service sectors (United Nations Development Programme, 2020). Similarly, the unabated COVID-19 situation coupled with the ongoing war in Ukraine with strong cascading effects on LMICs will increase vulnerabilities and push many more Gambians into extreme poverty (United Nations, 2020a; 2022). To address these challenges, the government should design an iterative and progressive contribution formula using means testing approach to fix premium rates. Furthermore, the government should explore other revenue sources such as indirect taxes, which are generally considered progressive to fund the scheme.

The discrepancy between significance levels for household income in the two models could be attributed to starting point bias. In the linear regression analysis, respondents stated their maximum amounts to pay for NHIS as opposed to the DBDC model, where respondents were asked to respond to three bids presented to them. Our finding showed that after adjusting for the annual household income, the majority of the respondents fall below the poverty line. This could be attributed to changes in the ordering of individuals by income as a result of dividing household income by an equivalence scale. It is important to note that our study was interested in adjusting the household’s annual disposable income relative to household size. In contrast, GBoS whose national poverty estimates we highlighted in Introduction used the Foster–Greer–Thorbecke class of decomposable poverty measure comprising headcount ratio, the poverty gap index (depth of poverty) and the poverty severity index (the squared poverty gap). Using a different poverty estimation approach to GBoS’s has a tendency to affect our poverty estimates and we discussed this below.

The WTP estimated from our study has policy implication for NHIS implementation particularly on enrolment and financial sustainability of the scheme. Given that high WTP does not equal ability to pay, decision-makers at the National Health Insurance Authority should learn from the experiences of Ghana and Kenya during the early phase of their NHIS implementation. Evidence from these countries, which have similar features to Gambia’s NHIS, showed that enrolment was somewhat high from the outset but plateaued and/or dropped over time (Maina *et al.*, 2016; Kotoh *et al.*, 2018). Few studies attributed this to cost, poor quality and a far too generous benefit package that was difficult to sustain financially (Maina *et al.*, 2016; Duku, 2018; Otieno *et al.*, 2019). In view of this, decision-makers should establish an affordable and progressive premium contribution rate and develop an explicit benefit package that offers quality and can be sustainably financed with heavy reliance on domestic revenue.

### Limitations

One of the criticisms of DBDC with the follow-up approach is its inherent starting point bias in the measurement of WTP. Although we employed measures to reduce this bias as much as possible, the first bid appeared relatively high compared with other WTP studies. The most appropriate approach would have been to undertake an in-country estimation as opposed to using the mean GDP per capita of nine WTP studies conducted in Western Africa. Another criticism of this approach is the hypothetical bias. Respondents may not recall the events they experienced in the preceding year and may not objectively respond to the hypothetical NHIS scenario. It was difficult to adjust household income in our study using GBoS poverty estimation. Our decision to apply the equivalence scale to adjust household income relative to household size pushed many of our respondents below the poverty line. However, we applied a rigorous probability proportional to size sampling technique using adequate sample size with >90% response rate. We believe that the difference in household income reported in our study relative to GBoS’s finding was not due to sampling bias, but rather because of limitations of income measurement in our study.

## Conclusion

Our study has shown that the majority of Gambians have indicated their willingness to join and pay for NHIS with an average WTP value of US$23.27 (GMD1119.82). Results of the two models together have shown that gender, level of education and household income influenced Gambians’ WTP and maximum amount to pay for NHIS. Despite the strong public support for NHIS, the high poverty and unemployment rate are threats to the sustainability of the scheme. In view of this, the government should increase domestic revenue-raising capacity to respond to the funding needs of the scheme consistently and predictably. In response to these findings, the government has a policy window to implement a sustainable NHIS that can propel Gambia towards UHC. Policymakers should also consider factors that influence Gambians’ WTP and used means testing approach when setting contribution rates and exemption criteria.

## Supplementary Material

czac089_Supp

## Reflexivity statement

The authors include researchers from low- and middle-income countries (LMICs), middle-income country and high-income country and include a female with multiple levels of seniority. All authors have extensive experience conducting research in LMICs and have strong background in health economics, health financing, health policy and systems research in global North/South. The lead author is a national of The Gambia, where the study was conducted.

a) WTP for a National Health Insurance Scheme (NHIS) in The Gambia.
b) Universal health coverage (UHC), health financing, health insurance scheme and willingness to pay (WTP).

a) Many LMICs including The Gambia have initiated health financing reforms with a view to making progress towards UHC.
b) The Gambia National Health Insurance Act, enacted into law in 2021, established an NHIS that will pay the healthcare cost of its members.
c) Our results showed that many Gambians have expressed their WTP for NHIS in return for improved health services.
d) Our findings can inform policymakers in The Gambia and other sub-Saharan African countries when establishing contribution rates and exemption criteria during social health insurance scheme implementation.

a) Abstract: 281 words.
b) Full article: 5259 words.

This study was granted ethical approval by the following institutions:

a) The Gambia Government/Medical Research Council Gambia Joint Ethics Committee granted ethical approval for this research (R018026v4.1).
b) Norwegian Centre for Research Data granted data processing ethical approval for this research (562 557).
c) Norwegian Research Committee for Medical and Health Research Ethics exempted this research from ethical review (2018/1891).
