# Supplementary material for: Willingness to pay for a National Health Insurance Scheme in The Gambia: a contingent valuation study
Source: Health Policy Plan. 2022 Oct 27;38(1):61–73. doi: 10.1093/heapol/czac089 (PMC9849717; doi:10.1093/heapol/czac089)
Supplement: czac089_Supp [file czac089_supp.zip › Supplement_DBDC_GLM_equation.docx]

**Supplement to: Willingness to pay for a national health insurance scheme in The Gambia: A contingent valuation study.**

**Details on the Double- Bounded Dichotomous Choice and Linear Regression Models**

Part 1: Double-Bounded Dichotomous Choice with follow up Model

We model willingness to pay (WTP) as a linear function $WTP_{i}\left( z_{i}, u_{i} \right)=z_{i}\beta+u_{i},$ where $z_{i}$ is a vector of explanatory variables, $\beta$ is a vector of parameters, and $u_{i}$ is an error term assumed to be independent and randomly distributed with mean zero and a constant variance, $\sigma^{2}$. Let the first bid amount be denoted $t_{1}$ and the second amount $t_{2}$, and then each individual will be in one of the following categories:

1. If the individual answers *yes* to the first question and *no* to the second question, then $t_{2}>t_{1}$. In this case, we can infer that $t_{1}\leq WTP\leq t_{2}$.
2. If the individual answers *yes* to the first question and *yes* to the second one, then $t_{i}\leq WTP<\infty$.
3. If the individual answers *no* to the first question and *yes* to the second one, then $t_{2}<t_{1}$. In this case, we have $t_{2}\leq WTP<t_{1}$.
4. If the individual answers *no* to the first and second questions, then we have $0<WTP<t_{2}$.

Thus, the probability of each of the four scenarios are defined below:

1. $P_{r}(t_{1}$≤ WTP < $t_{2}$) = $\left( \frac{t_{1}- z_{i}^{'}\beta}{\sigma}\leq\frac{u_{i}}{\sigma}< \frac{t_{2}-z_{i}^{'}\beta}{\sigma} \right)$ = $\Phi$ $\left( z_{i}^{'}\frac{\beta}{\sigma}- \frac{t_{1}}{\sigma} \right)$ - $\Phi$ $\left( z_{i}^{'}\frac{\beta}{\sigma}- \frac{t_{2}}{\sigma} \right)$
2. $P_{r}(WTP>t_{1},$ WTP > $t_{2}$) = $\Phi$ $\left( z_{i}^{'}\frac{\beta}{\sigma}- \frac{t_{2}}{\sigma} \right)$
3. $P_{r}(t_{2}$≤ WTP < $t_{1}$) = $\Phi$ $\left( z_{i}^{'}\frac{\beta}{\sigma}- \frac{t_{2}}{\sigma} \right)$ - $\Phi$ $\left( z_{i}^{'}\frac{\beta}{\sigma}- \frac{t_{1}}{\sigma} \right)$
4. $P_{r}(WTP<t_{1},$ WTP < $t_{2}$) = 1 - $\Phi$ $\left( z_{i}^{'}\frac{\beta}{\sigma}- \frac{t_{2}}{\sigma} \right)$

To directly obtain estimates for $\beta$ and $\sigma$ using the maximum likelihood method, the function should be maximized to find the parameters of the model as shown below:

$$\sum_{i=1}^{N} \left[ \begin{aligned} d_{i}^{yn}\ln\left( \Phi\left( z_{i}^{'}\frac{\beta}{\sigma}- \frac{t_{1}}{\sigma} \right)- \Phi\left( z_{i}^{'}\frac{\beta}{\sigma}- \frac{t_{2}}{\sigma} \right) \right)+ d_{i}^{yy}\ln\left( \Phi\left( z_{i}^{'}\frac{\beta}{\sigma}- \frac{t_{2}}{\sigma} \right) \right) + \\ d_{i}^{ny}\ln\left( \Phi\left( z_{i}^{'}\frac{\beta}{\sigma}- \frac{t_{2}}{\sigma} \right)- \Phi\left( z_{i}^{'}\frac{\beta}{\sigma}- \frac{t_{2}}{\sigma} \right) \right)+ d_{i}^{nn}\ln\left( 1-\Phi\left( z_{i}^{'}\frac{\beta}{\sigma}- \frac{t_{2}}{\sigma} \right) \right) \end{aligned} \right]$$

Part 2: Linear Regression Model (Generalized Linear Model)

Maximum amount to pay = $\beta_{0}$ + $\beta_{1}$ Gender + $\beta_{2}$ Age +$\beta_{3}$ Education + $\beta_{4}$ Household Size + $\beta_{5}$ Household Income + $\beta_{6}$ Hospitalization + $\beta_{7}$ State of Health
